# A model for the role of gut bacteria in the development of autoimmunity for type 1 diabetes

**DOI:** 10.1007/s00125-015-3614-8

**Published:** 2015-05-10

**Authors:** Austin G. Davis-Richardson, Eric W. Triplett

**Affiliations:** Microbiology and Cell Science Department, Institute of Food and Agricultural Sciences, 1355 Museum Road, PO Box 110700, Gainesville, FL 32611-0700 USA

**Keywords:** Bacteroidetes, DNA methylation, Gut bacteria, Gut microbial community, Review, Short-chain fatty acids

## Abstract

Several lines of evidence suggest a role for the gut microbiome in type 1 diabetes. Treating diabetes-prone rodents with probiotics or antibiotics prevents the development of the disorder. Diabetes-prone rodents also have a distinctly different gut microbiome compared with healthy rodents. Recent studies in children with a high genetic risk for type 1 diabetes demonstrate significant differences in the gut microbiome between children who develop autoimmunity for the disease and those who remain healthy. However, the differences in microbiome composition between autoimmune and healthy children are not consistent across all studies because of the strong environmental influences on microbiome composition, particularly diet and geography. Controlling confounding factors of microbiome composition uncovers bacterial associations with disease. For example, in a human cohort from a single Finnish city where geography is confined, a strong association between one dominant bacterial species, *Bacteroides dorei*, and type 1 diabetes was discovered (Davis-Richardson et al. *Front Microbiol*^2014^;5:678). Beyond this, recent DNA methylation analyses suggest that a thorough epigenetic analysis of the gut microbiome may be warranted. These studies suggest a testable model whereby a diet high in fat and gluten and low in resistant starch may be the primary driver of gut dysbiosis. This dysbiosis may cause a lack of butyrate production by gut bacteria, which, in turn, leads to the development of a permeable gut followed by autoimmunity. The bacterial community responsible for these changes in butyrate production may vary around the world, but bacteria of the genus *Bacteroides* are thought to play a key role.

## Introduction

The incidence of type 1 diabetes in many developed countries has been increasing at rates faster than can be explained by the known genetic propensity towards the disease [1]. The environmental triggers of this disease have not yet been identified, despite the many efforts to associate diet, vitamin D, viruses and other factors with disease [1]. A leaky gut has been correlated with type 1 diabetes [2], and an aberrant gut microbiome was proposed as the factor that results in a leaky gut followed by altered immune responses leading to disease [3]. This review describes a model for the role of the gut microbiome in type 1 diabetes based on the latest results.

### Animal models

Past work with non-obese diabetic (NOD) mice and BioBreeding diabetes-prone (BB-DP) rats provided the first evidence that bacteria may play an important role in the onset of type 1 diabetes. BB-DP rats fed sulfamethoxazole, trimethoprim and colistin sulphate demonstrate a significantly decreased incidence of diabetes compared with controls [4]. A similar increase in disease-free animals vs controls was observed in NOD mice after administration of doxycycline or vancomycin [5, 6]. *Bacteroides* were higher in stools from the BB-DP rat while *Lactobacillus* was higher in the diabetes-resistant rat (BB-DR) [7]. Strains of *Lactobacillus johnsonii* and *Lactobacillus reuteri* isolated from BB-DP rats prevented and promoted diabetes in BB-DP rats, respectively [8]. The *L. johnsonii* strain induces a T helper 17 cell bias in the mesenteric lymph nodes of C57BL/6 mice while the *L. reuteri* strain does not [9].

Although the incidence of insulitis is not significantly different between germ-free and specific-pathogen-free (SPF) NOD mice, germ-free mice develop insulitis earlier than SPF mice [10, 11]. The presence of segmented filamentous bacteria (SFB) in female NOD mice is correlated with decreased insulitis incidence and with the percentage of IL-17-expressing lymphocytes [12].

Diet is known to significantly affect diabetes incidence in NOD mice. For example, a high-cereal diet increases diabetes incidence in NOD mice, while a high-protein diet reduces risk [13]. Recent studies observed that acidic drinking water also increases diabetes incidence in NOD mice, and this is prevented by inoculating mice with SFB [14].

### Humans

The results of the murine experiments encouraged efforts to determine whether associations between gut bacteria and type 1 diabetes could be discovered in humans. Rodent model investigations suggested that healthy children may have high populations of probiotic-like bacteria such as *Lactobacillus,* while the gut microbiome of unhealthy children may be dominated by *Bacteroides*. Perhaps antibiotic or probiotic use early in life could prevent type 1 diabetes, but this requires more study. Analysis of a Finnish dataset revealed no connection between antibiotic use and autoimmunity for type 1 diabetes [15], but larger cohorts may be needed to observe an antibiotic influence on this disease. Early on, a simple picture was expected to emerge by simply studying the stool content of a few human cohorts with individuals at high genetic risk of type 1 diabetes.

At first, with very small human cohorts with only four healthy children and four children with type 1 diabetes autoantibodies from the Diabetes Prediction and Prevention (DIPP) study in Finland, the human situation did appear to be very similar to the murine one [16, 17]. These studies showed highly significant taxonomic and functional differences between cases and controls prior to autoimmunity for type 1 diabetes. Levels of *Bacteroides* were higher in children with autoimmunity, and levels of seemingly protective unclassified Firmicutes were higher in healthy children. These results match the findings of the murine experiments except for the fact that the human samples had negligible amounts of *Lactobacillus*.

Contrasting results were obtained from an investigation of 298 stool samples collected during the first 3 years of age from 22 case and 22 control children enrolled in the German BABYDIET study [18]. Unlike the small Finnish cohort, no significant differences between taxa were observed between cases and controls after correction for false discovery rate. Instead, network analysis showed that bacterial communities in cases were far less strongly correlated with each other at <6 months of age and again at about 2 years of age than in controls. However, the results from the Finnish and German cohorts were similar in that the autoimmune bacterial communities were less stable than the healthy communities. The cause for this instability in autoimmune samples remains unknown.

A still larger set of 947 stool samples collected during the DIPP study from 29 cases and 47 controls during the first 4 years of life was examined [15]. All of the children enrolled in the DIPP study are genetically at high risk for type 1 diabetes. Analysis of these samples revealed a very striking result: approximately 8 months prior to the average time of appearance of the first autoantibody in cases, a large increase in one bacterial species, *Bacteroides dorei*, was observed. In addition, *B. dorei* was by far the most abundant species in autoimmune prone children, with over 20% of the population represented by this one species—a rate that was more than double the relative abundance of this species in healthy children. This difference was highly statistically significant even after correcting for false discovery rate and was found to occur at about the same time as the introduction of solid food. A few other minor taxonomic differences were also observed, but nothing as striking as the *B. dorei* result.

So why was the *B. dorei* result seen in the large Finnish DIPP cohort but not in the German BABYDIET study? At first glance, it may appear that since the *B. dorei* result in Finland was not reproduced in Germany, it must then be an anomaly. However, there is one very important difference between the designs of these two cohorts that may explain this difference. The samples used in the DIPP study were all from children born in the same hospital, Turku University Hospital [15]. All of the children lived within 80 km of each other. Thus, many of the known confounders of the microbiome community were controlled; that is, climate, diet, culture, water supply, pollution levels, air quality and medical practices were all very similar for these children.

In contrast, the children in the BABYDIET cohort were from all over Germany, allowing enough variability in the microbiome confounders to affect microbiome composition. In addition, all of the children in the BABYDIET study had first-degree relatives with type 1 diabetes, while in DIPP the children were chosen for the study based on their high-risk HLA genotype at birth. These confounders add to the noise of the data and may explain why no significant taxonomic differences were observed between cases and controls. These confounders may have been sufficient to mask any taxonomic differences that might otherwise have been detected in the BABYDIET study but too weak to mask the network differences seen in this cohort [18].

Hence, a working hypothesis is that geography plays a very large role in whether it is possible to observe significant case–control differences in any cohort designed to examine associations between the microbiome and disease. From this perspective, the microbiome results from the BABYDIET and DIPP cohorts do not contradict each other but rather help us understand how best to design cohorts for future disease-related microbiome studies.

Two other recent studies from Europe and Mexico showed taxonomic differences in the gut microbiome between individuals with type 1 diabetes and individuals without the disease [19, 20]. In both studies, levels of *Bacteroides* were significantly higher in those children with type 1 diabetes compared with healthy children. These studies were intended to show the effect of the disease itself as opposed to identifying a trigger for autoimmunity as in the previous studies. The Mexican study was restricted to Sonora state, which borders Arizona, and was limited to 29 children, including eight controls. Based on the findings of these studies and the pre-autoimmunity cohorts described above, *Bacteroides* appears to be a major contributor to microbiome dysbiosis both prior to the development of autoimmunity and after disease diagnosis.

There is evidence that the human genome has some control over the taxonomic composition of bacteria in the gut. However, it is not known whether a genetic propensity to type 1 diabetes, as manifested by HLA genotype, affects bacterial gut composition [21]. The studies conducted to date on the relationship between type 1 diabetes autoimmunity and the microbiome have not seen an HLA genotype effect on the microbiome, but these studies did not have enough participants to observe such an effect [15, 18].

The hygiene hypothesis suggests that a lack of exposure to microbes early in life under hygiene conditions in the developed world contributes to a weakened immune system incapable of warding off the effects of detrimental bacteria in the gut [22]. Efforts are underway to examine this issue carefully between Finland and neighbouring Estonia and Russian Karelia [22]. Testing this hypothesis will be difficult in any study protocol. Our view that separating signal from noise is best done by careful cohort design intended to reduce the confounders of the microbiome.

## Approaches to reduce these confounders in cohort design

Our recent results suggest that striking case–control differences can be observed when the geographic distribution of a high-risk cohort is limited to one city and its immediate surroundings. Another issue is whether type 1 diabetes risk is higher in urban or rural areas [23]. Differences in large gut microbiome taxa and alpha diversity differences were recently observed across six clinical sites in children at high genetic risk for type 1 diabetes [24]. Higher socioeconomic status is typically associated with type 1 diabetes and other autoimmune diseases [25]. To date there are no reports in the literature on socioeconomic status and gut microbiome composition, but socioeconomic status has been shown to be associated with the oral microbiome [26]. However, given that geography and diet are well known to affect gut microbiome composition and that socioeconomic status affects diet and place of residence, cohort design should take these factors into account or in some way control for them. To date, no clear associations between type 1 diabetes and diet have been made, but this is being carefully examined by The Environmental Determinants of Diabetes in the Young (TEDDY) study group [27].

An ideal cohort design might be one that includes samples from a set of cities similar to Turku where the cohort includes children all born in the same hospital and all living in the surrounding area. The variability of other environmental factors, such as climate, drinking water, health practices and air quality, are similar across a single city. Having a set of such cities each with children at high risk might allow us to identify bacteria or bacterial functions that are associated with autoimmunity for type 1 diabetes across a broad geographic scale.

Sampling from families may be a means of reducing the noise in the microbiome data. That is, rather than collecting samples from healthy controls matched for genetic risk, control samples could be obtained from healthy siblings. But age matching is important, so noise could be further controlled by enrolling identical and non-identical twins for the study of those at high genetic risk for the disease at birth. This would be a difficult undertaking in a small city such as Turku but might work well in a much larger city in the same country, such as Helsinki.

Another way of reducing noise would be to determine the changes that can occur in the gut microbiome communities in stools in response to long-term exposure to room temperature and air. Approaches must be developed that make it easier for families to collect stools in a manner that allows for rapid freezing. Microbial communities in stools do change significantly after 24 h of exposure to air, and these changes include a sharp decline in the relative abundance of *Bacteroides* strains [28], which can survive air but do not divide under aerobic conditions. To date, none of the studies to investigate the microbiome of children at high genetic risk for type 1 diabetes has been designed with the microbiome in mind. In addition to the aforementioned improvements in study design and larger cohorts with limited confounding, advancements in big data analysis methods should also help to facilitate within- and between-study analyses.

## Does the *B. dorei* genome suggest functions associated with the development of autoimmunity for type 1 diabetes?

As described above, a recent study showed a strong association between high levels of *B. dorei* in the gut several months prior to the appearance of the first type 1 diabetes autoantibody [15]. A first step towards determining whether *B. dorei* causes type 1 diabetes autoimmunity would be to culture it from the Turku DIPP samples, then inoculate NOD mice with this organism and determine whether this strain(s) reduces the survival rates of the mice. Although this is a high-risk strategy and not without significant intellectual challenges, it would be worthwhile to conduct such an experiment because even though *B. dorei* may not be involved in type 1 diabetes onset in other populations, a certain set of functions expressed by *B. dorei* may have wide applicability to autoimmune disease around the globe.

As more bacterial metagenomes are obtained from stool samples of high-risk children, quantifying the relative abundance of homologues or paralogues of *B. dorei* genes will be an easy task. Additional *Bacteroides* species may be associated with type 1 diabetes autoimmunity in other cohorts, which will allow us to build a database of genes in common with all *Bacteroides* associated with diseases vs those genes in *Bacteroides* species not associated with disease. A similar approach can be used on bacterial species or strains thought to be protective against the disease. Bacterial functions associated with disease have been listed for a very small cohort from the DIPP study [16]. Further studies are warranted on an increased number of individuals and more samples across additional time points.

## Is diet the source of high levels of *Bacteroides* associated with type 1 diabetes autoimmunity?

As *Bacteroides* have been associated with type 1 diabetes autoantibodies, the next obvious question pertains to the source of these bacteria. That is, if a single species or set of closely related species is associated with type 1 diabetes, the need to hunt for more bacterial associations with type 1 diabetes declines and the problem becomes one of searching for those environmental factors that increase the abundance of these *Bacteroides* species in the gut. In the stool samples of autoimmune Turku children, levels of *B. dorei* peak at about 7 months of age, about 1 month prior to the Finnish national standard for the timeframe of solid food introduction [15]. If the early introduction of solid food is related to raised *Bacteroides* levels, which foods are known to increase *Bacteroides* abundance in the gut?

High-fat and high-gluten diets encourage colonisation of the gut by *Bacteroides. Bacteroides* populations in the human gut have been strongly correlated with a diet high in protein, animal fat, cholesterol and other fats [29]. *Bacteroides* are well known as the primary proteolytic bacteria in the gut [30]. Not surprisingly, vegans and vegetarians have low levels of *Bacteroides* in their stools [31]. To date, connections between a vegetarian diet and the progression towards type 1 diabetes have not been studied.

Examination of the gut microbiome in coeliac disease suggests that gluten may play a role in *Bacteroides* abundance. The prevalence of *B. dorei* was 67% higher in children with active coeliac disease compared with the same children on a gluten-free diet for two years [32]. Similarly, formula-fed children at high risk for coeliac disease were approximately six-times more likely to possess *B. dorei* in stool than low-risk controls [33]. These results encourage further work to determine whether gluten increases population growth of *B. dorei* in children at high risk for autoimmunity. Resistant starch may also play a significant role in preventing type 1 diabetes as it has been reported to increase faecal butyrate levels [34].

## Association between gut integrity and type 1 diabetes in humans

The onset of type 1 diabetes might involve an aberrant intestinal microbiome creating the conditions for gut leakiness followed by mucosal immune responses that lead to an attack of insulin-producing islet cells [3]. Consistent with this notion, type 1 diabetic individuals have been shown to have a leakier gut than controls [35–37], and it has been reported that this leakiness is not caused by type 1 diabetes, rather, type 1 diabetes is preceded by a leaky gut [2]. In the NOD mouse model, a leaky gut can lead to the activation of diabetogenic CD8^+^ cells which, in turn, lead to the development of insulitis [38]. Mechanisms for a connection between the leaky gut and type 1 diabetes have been reviewed previously [39, 40].

## A role for short-chain fatty acids and gut integrity

Butyrate is one of the short-chain fatty acids (SCFAs) produced by bacterial fermentation in the gut. The ratio of butyrate:acetate:proprionate in the gut varies widely, but these three SCFAs make up the majority of the SCFA content in this organ. The concentration of SCFAs in the gut can be as high as 140 mmol/l and these SCFAs can provide up to 10% of the daily human caloric needs [41]. Butyrate reduces trans-membrane transport of *Escherichia coli* across confluent monolayers of colon-derived cell lines [42]. In addition, inflammation may reduce butyrate transport through the downregulation of a butyrate transporter [43].

Butyrate is also known to play two important roles in maintaining the integrity of the epithelial layer. First, butyrate induces the expression of mucin-producing genes in the epithelial layer [44]. Mucin is a host glycoprotein that protects the epithelial layer from attack by bacteria or bacterial metabolites. Second, butyrate restores and maintains tight junction assembly in epithelial layers [45, 46]. These tight junctions are formed between epithelial cells and serve as gatekeepers for metabolite transport across the epithelium. Other SCFAs do not induce tight junction formation or mucin synthesis.

Healthy children have a higher proportion of butyrate-producing gut bacteria in their microbiomes compared with children expressing at least one beta islet cell autoantibody [16, 47]. Based on this, the ratio of butyrate-producing to other SCFA-producing bacteria in the gut was proposed to be a key regulator in determining the gut health of a child at high risk for type 1 diabetes [16]. However, there is no evidence to date linking butyrate levels in the gut to the progression of diabetes in either animals or humans.

## Can gut bacteria regulate the epigenome of the human epithelial cell?

Butyrate has roles other than maintenance of the integrity of the gut epithelial layer. In particular, there is increasing evidence that butyrate has epigenetic effects that may be very important in type 1 diabetes, as unknown environmental triggers must be playing a role in the rapid increase in the incidence of this disease [48].

Butyrate induces the methylation of promoter regions, which causes both up- and downregulation in different sets of human genes [49]. Histone acetylation also appears to be regulated by butyrate production. Butyrate reduces lipopolysaccharide-induced inflammation in the intestine through modulation of antioxidant defence systems [50], nitric oxide production, and expression of inflammatory cytokines [51]. Many of these effects appear to be modulated by the inhibition of histone deacetylases in macrophages [51]. Also, histone acetylation in the promoter of the *Foxp3* locus induces the differentiation of regulatory T cells in mice [52]. Several epigenetic changes in host epithelial cells may be caused by increased butyrate production from probiotic strains [53].

## Is there a role for the methylome of the gut microbiome in the development of type 1 diabetes autoimmunity?

The genomes of two of the *B. dorei* strains from the DIPP Turku cohort have been sequenced to completion and, physiologically, appear to be typical *Bacteroides* in that they metabolise a broad spectrum of compounds and have many antibiotic-resistance genes [54]. One of these genomes was derived from a child who later developed type 1 diabetes-associated autoantibodies (case) while the other genome was from a high-risk healthy child (control). The case genome includes a bacteriophage with a DNA adenine methyltransferase gene that appears to be responsible for the methylation of all but three of the 20,554 GATC motifs in the genome [54]. Such phage-borne DNA methyltransferases have been found in several bacteria [55]. None of the nearly 19,000 GATC motifs in the control genome were methylated, presumably because it lacked this phage and its orphan DNA methyltransferase gene. This work was the first report of DNA methylation differences within a bacterial species in the microbiome [54].

Although these methylation results from two *B. dorei* genomes cannot be associated with disease, they suggest that a wider examination of methylation patterns in more samples is warranted. DNA methylation patterns are known to affect gene expression in bacteria [56]. They control epigenetic responses that are often regulated by environmental exposure. Indeed, a few methylation sites in the human genome have been associated with type 1 diabetes pathogenesis [57, 58]. It is therefore conceivable that methylation differences in the genomes of bacteria associated with type 1 diabetes may play a role in disease.

## Conclusions

**Fig. 1:**
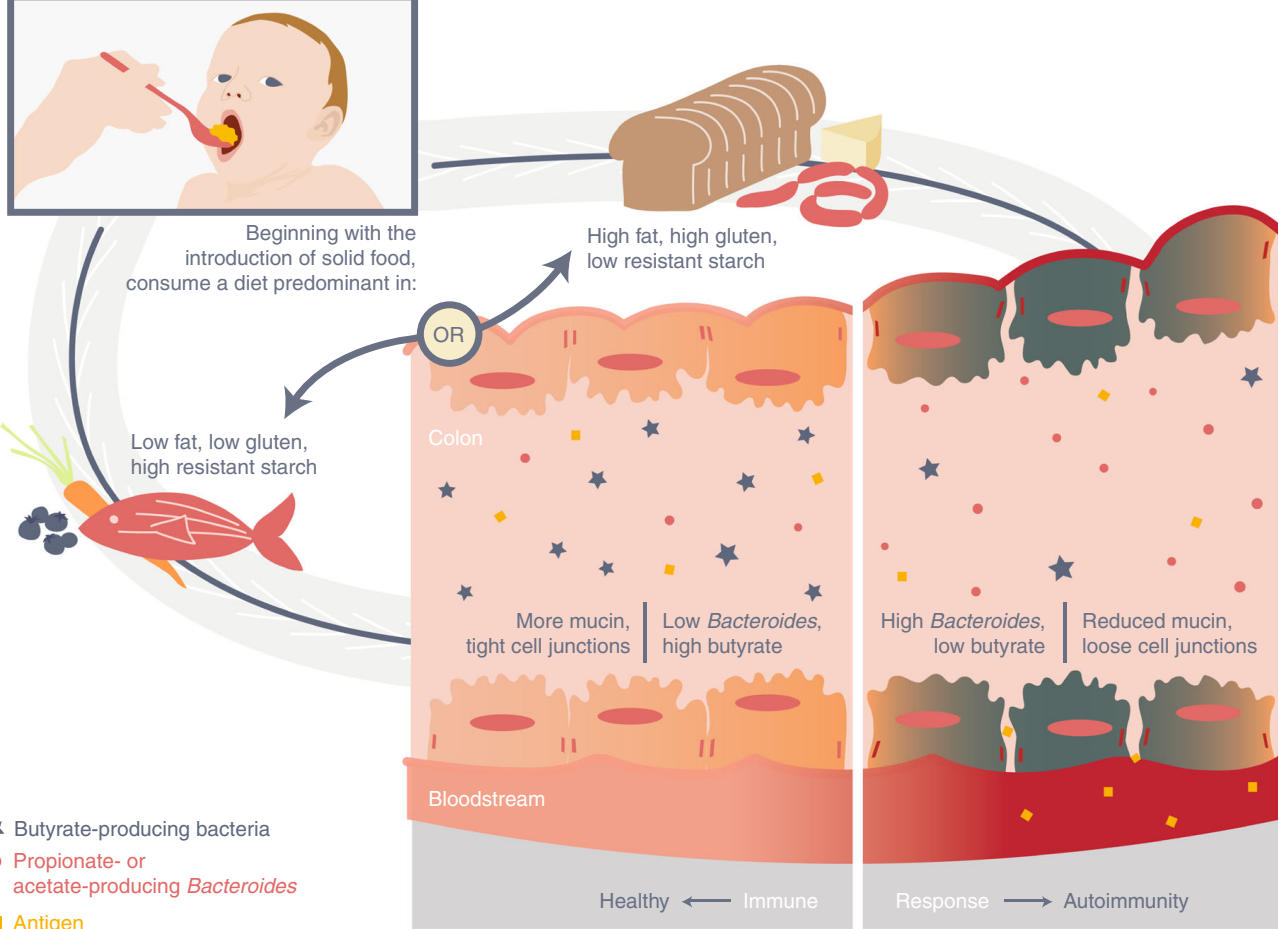
Model for the development of autoimmunity for type 1 diabetes based on a diet-induced disproportionate high number of *Bacteroides* in the gut, leading to a leaky gut and autoimmunity for the disease

The analyses described above suggest a revised model for the role of the microbiome in type 1 diabetes, one that is based on a previous model [16] but takes into account the role of diet (Fig. 1). In this revised model, type 1 diabetes autoimmunity occurs when a high-fat, high-gluten diet, perhaps one with a low level of resistant starch, increases *Bacteroides* colonisation in the gut. These bacteria ferment glucose to SCFAs other than butyrate. The lack of butyrate production reduces the likelihood of an intact gut epithelial layer. The resulting permeability causes bacterial antigen exposure that induces immune responses leading to type 1 diabetes autoimmunity.

Recent results suggest that the many confounders of gut microbiome composition need to be considered in cohort design for the optimal discovery of associations between the gut microbiome and disease. A cohort carefully designed to control for geography and diet as much as possible can eliminate much of the noise that is often associated with microbiome data. Future work will require experiments or observational studies that directly test each point in the model (Fig. 1).

## Abbreviations

BB-DP: BioBreeding diabetes-prone
BB-DR: BioBreeding diabetes-resistant
DIPP: Diabetes Prediction and Prevention
SCFA: Short-chain fatty acid
SFB: Segmented filamentous bacteria
SPF: Specific-pathogen-free
